# Supplementary material for: Identification of candidate mitochondrial inheritance determinants using the mammalian cell-free system
Source: eLife. 2023 Jul 20;12:RP85596. doi: 10.7554/eLife.85596 (PMC10393022; doi:10.7554/eLife.85596)
Supplement: Supplementary file 2. [file elife-85596-supp2.docx]

**Supplementary file 2:** Proteomic identification of mitophagy and sperm remodeling cofactors in the porcine cell-free system after 4 hours of co-incubation.

| Protein of Interest  Legend:  GN = Gene name  PE = protein existence level  SV = Sequence version  (PE and SV only included for proteins which had multiple versions in the table) | Proposed role in autophagy/mitophagy/post-fertilization sperm structure remodeling by zygote, and original references describing identified proteins’ role in those events. | P value based on 3 rep T-test analysis |
| --- | --- | --- |
| **CLASS 1 – Proteins detectable on spermatozoa only after extract exposure.** | | |
| Metalloendopeptidase a.k.a. Ovastacin GN: ASTL | Member of peptidase family involved in reproductive protein processing in *Drosophila;* (ov)astacin is secreted by mouse oocyte to cleave zona, prevent polyspermy. Ram *et al.,* 2006 DOI: [10.1073/pnas.0606228103](https://doi.org/10.1073/pnas.0606228103) ; Burkart *et al.,* 2012; DOI: [10.1083/jcb.201112094](https://doi.org/10.1083/jcb.201112094) | 0.00003 |
| Zona pellucida sperm-binding protein 3 GN: ZP3 (PE: 1, SV: 1) | Involved in sperm-egg recognition, acrosomal reaction, and polyspermy defense. Zimmerman *et al.,* 2011 DOI: [10.1371/journal.pone.0017256](https://dx.doi.org/10.1371%2Fjournal.pone.0017256) | 0.00003 |
| Major vault protein GN: MVP | Regulates cell growth and survival through the STAT3 and Akt signaling pathways. Das *et al.,* 2016 DOI: [10.1016/j.cellsig.2015.10.007](https://doi.org/10.1016/j.cellsig.2015.10.007) | 0.00006 |
| Peptidyl arginine deiminase 6 GN: PADI6 | Essential for early zygotal development, specifically cytoskeletal sheet formation through citrullination. Essential for female fertility. Esposito *et al.,* 2007 DOI: [10.1016/j.mce.2007.05.005](http://doi.org/10.1016/j.mce.2007.05.005) | 0.00007 |
| Malic enzyme GN: ME2 | Participates in pyruvate metabolic process, located in the mitochondria. www.unitprot.org | 0.00008 |
| Aprotinin Accession: B0LXF7 | A serine protease inhibitor which decreases bleeding. Robert *et al.,* 1996 DOI: [10.1177/106002809603000410](https://doi.org/10.1177/106002809603000410) | 0.0001 |
| Zona pellucida sperm-binding protein 4 GN: ZP4 | Involved in sperm-egg recognition, acrosomal reaction, and polyspermy defense. Zimmerman *et al.,* 2011 DOI: [10.1371/journal.pone.0017256](https://dx.doi.org/10.1371%2Fjournal.pone.0017256) | 0.0002 |
| Uncharacterized protein GN: PRDX3 | Necessary for normal mitochondrial function. Localizes to the mitochondria after peroxide exposure and acts as a peroxide reducing agent. Wonsey *et al.,* 2002 DOI: [10.1073/pnas.102523299](https://doi.org/10.1073/pnas.102523299) | 0.0006 |
| Zona pellucida sperm-binding protein 3 GN: ZP3 (PE: 4, SV: 1) | Involved in sperm-egg recognition, acrosomal reaction, and polyspermy defense. Zimmerman *et al.,* 2011 DOI: [10.1371/journal.pone.0017256](https://dx.doi.org/10.1371%2Fjournal.pone.0017256) | 0.1835 |
| DNA (cytosine-5)-methyltransferase GN: DNMT1 | Helps maintain DNA methylation problems through cell divisions. It is found within the cytoplasm of MII oocytes and early-stage embryos, later localizing to the nuclei of 8 – 16 cell embryos. Lodde *et al.,* 2009 DOI: [10.4081/ejh.2009.e24](https://doi.org/10.4081/ejh.2009.e24) | 0.1835 |
| Nucleophosmin/nucleoplasmin 2 GN: NPM2 | Sperm nucleus remodeling after fertilization. Required for histone import into paternal pronucleus. McLay & Clark., 2003 DOI: [10.1530/rep.0.1250625](https://doi.org/10.1530/rep.0.1250625) | 0.1836 |
| Zona pellucida sperm-binding protein 2 GN: ZP2 (SV:2) | Involved in sperm-egg recognition, acrosomal reaction, and polyspermy defense. Zimmerman *et al.,* 2011 DOI: [10.1371/journal.pone.0017256](https://dx.doi.org/10.1371%2Fjournal.pone.0017256) | 0.1837 |
| 1-aminocyclopropane-1-carboxylate synthase homolog (inactive) like GN: ACCSL | Binds pyridoxal phosphates and has catalytic activity. www.uniprot.org | 0.1842 |
| Extended synaptotagmin 1 GN: ESYT1 | Located in the ER and acts as a lipid transport protein. Interacts with PI(4,5)P, and calcium to transfer lipids through membranes. Xie *et al.,* 2018 DOI: [10.1096/fj.201801878R](https://doi.org/10.1096/fj.201801878r) | 0.1859 |
| **CLASS 2 - Detected in the vehicle and primed control spermatozoa, increased in extract-exposed spermatozoa.** | | |
| Peroxiredoxin 2 GN: PRDX2 (SV: 1) | Antioxidant enzyme that plays role in ROS protection immediately after fertilization. Morita *et al.,* 2018 DOI: [10.1262/jrd.2018-005](https://doi.org/10.1262/jrd.2018-005) | 0.0004 |
| Cytochrome c1 GN: CYC1 | Part of the mitochondrial electron transport chain. Forms a complex with cytochrome c and cytochrome oxidase. Located on the inner mitochondrial membrane. Chiang & King 1979 PMID:33986 | 0.0125 |
| Cytochrome c oxidase subunit 5A GN: COX5A | Cytochrome c oxidase is a protein complex made up of 13 subunits encoded by mtDNA and nuclear DNA. Without COX5A, cytochrome c oxidase fails to form. Fornuskova *et al.,* 2010 DOI: [10.1042/BJ20091714](https://doi.org/10.1042/bj20091714) | 0.0143 |
| Proteasome subunit alpha type: PSMA3 & PSMA2 | Pharmacological inhibition of 20S core activity prevents sperm mitophagy in porcine zygote. Sutovsky *et al.,* 2003 DOI: [10.1095/biolreprod.102.012799](https://doi.org/10.1095/biolreprod.102.012799) | 0.0152 |
| Stomatin like 2 GN: STOML2 | Mitochondrial protein which forms microdomains within the inner membrane. It is a regulator of mitochondrial translation. Mitsopoulos *et al.,* 2017 DOI: [10.1371/journal.pone.0179967](https://doi.org/10.1371/journal.pone.0179967) | 0.0174 |
| Outer dense fiber of sperm tails 2 GN: ODF2 | Sperm flagellum and centrosomal protein. Play a critical role in embryo preimplantation, this role remains unknown. Salmon *et al.,* 2006 DOI: [10.1002/dvg.20241](https://doi.org/10.1002/dvg.20241) | 0.0199 |
| Uncharacterized protein GN: NDUFB5 | Part of the mitochondrial respiratory chain complex I assembly. Integral component of the mitochondrial inner membrane. www.uniprot.org | 0.0219 |
| A-kinase anchoring protein 4 GN: AKAP4 | Flagellated sperm motility, transmembrane receptor protein serine/threonine kinase signaling pathway, located in the sperm principal piece. Assists in capacitation and acrosome reaction. Ben-Navi *et al.,* 2016 DOI: [10.1038/srep37922](https://doi.org/10.1038/srep37922) | 0.0424 |
| RIB43A domain with coiled-coils 1 GN: RIBC1 | A protein which is overexpressed in high fertility bull sperm. However, its function is not known. Muhammad Aslam *et al.,* 2018 DOI: [10.1016/j.theriogenology.2018.06.021](https://doi.org/10.1016/j.theriogenology.2018.06.021) | 0.0438 |
| NADH:ubiquinone oxidoreductase subunit B9 GN: NDUFB9 | Part of the mitochondrial respiratory chain complexes, specifically Complex I. Mutations of this subunit cause CI deficiency. One of the subunits responsible for CI and CIII interactions. Wu *et al.,* 2016 DOI: [10.1016/j.cell.2016.11.012](https://doi.org/10.1016/j.cell.2016.11.012) | 0.0465 |
| Uncharacterized protein Accession: A0A287A4B9 | BPTI/Kunitz inhibitor domain-containing protein. Serine-type endopeptidase inhibitor activity. Gene name: PTI. www.uniprot.org | 0.049 |
| Testis-specific serine kinase 6 GN: TSSK6 | Involved in sperm chromatin condensation, and crucial for spermiogenesis. Heat shock protein 90 regulates TSSK6 and shields it from ubiquitin-mediated catabolism. Jha *et al.,* 2013 DOI: [10.1074/jbc.M112.400978](https://doi.org/10.1074/jbc.m112.400978) | 0.0497 |
| Uncharacterized protein GN: OAZ3 | OA3 is expressed specifically during spermiogenesis. Essential for rigid junction between the head and tail of spermatozoa. Tokuhiro *et al.,* 2009 DOI: [10.1371/journal.pgen.1000712](https://doi.org/10.1371/journal.pgen.1000712) | 0.0615 |
| NADH:ubiquinone oxidoreductase subunit B10 GN: NDUFB10 | Part of the mitochondrial respiratory chain complexes, specifically Complex I. One of the subunits which forms intramolecular disulfides within CI. Wu *et al.,* 2016 DOI: [10.1016/j.cell.2016.11.012](https://doi.org/10.1016/j.cell.2016.11.012) | 0.0694 |
| Chromosome 9 open reading frame 135 GN: C9orf135 | Down regulated during gametogenesis. Believed to encode a membrane protein which serves as a surface marker for undifferentiated embryonic stem cells. Zhou *et al.,* 2017 DOI: [10.1038/srep45311](https://doi.org/10.1038/srep45311) | 0.0705 |
| Tektin 2 GN: TEKT2 | Associates with and organizes the central spindle during mitosis. Durcan *et al.,* 2008 DOI: [10.1083/jcb.200711160](https://doi.org/10.1083/jcb.200711160) | 0.0736 |
| Uncharacterized protein GN: BANF1 | Contributes to embryonic stem cell self-renewal properties, preventing them from differentiating too quickly. Cox *et al.,* 2011 DOI: [10.1242/jcs.083238](https://doi.org/10.1242/jcs.083238) | 0.0771 |
| Chromosome 4 open reading frame 45 GN: C4orf45 | Uncharacterized function. www.uniprot.org | 0.0914 |
| Proteasome subunit alpha type GN: PSMA8 | A testis-specific proteasomal subunit found during spermatogenesis. Appears crucial for proper meiotic exit by the forming spermatids. Gomez-H *et al.,* 2019 DOI: [10.1371/journal.pgen.1008316](https://doi.org/10.1371/journal.pgen.1008316) | 0.092 |
| Tektin 1 GN: TEKT1 | Found in both the acrosome and flagella of spermatozoa. May participate in sperm head cytoskeletal roles or serve a function in the acrosome reaction. Oiki *et al,* 2014 DOI: [10.2108/zsj.31.101](https://doi.org/10.2108/zsj.31.101) | 0.0922 |
| Ferritin GN: FTH1 | Acts as an inhibitor of death domain-associated protein (Daxx), which serves as an inhibition of the Daxx apoptosis pathway. Liu *et al.,* 2012 DOI: [10.1007/s11033-011-0811-5](https://doi.org/10.1007/s11033-011-0811-5) | 0.0949 |
| **CLASS 3 - Detected in spermatozoa before extract exposure, reduced after extract exposure.** | | |
| Oxoglutarate dehydrogenase L GN: OGDHL | A rate-limiting component of the OGDH complex, a mitochondrial protein complex. OGDHL regulates cell growth, apoptosis and seems to play a role in cancer development when overexpressed. Sen *et al.,* 2012 DOI: [10.1371/journal.pone.0048770](https://doi.org/10.1371/journal.pone.0048770) | 0.0008 |
| Kinesin-like protein GN: KIF5B | Involved in centrosome localization, cytoplasmic organization and assists with regulating protein localization to the plasma membrane. www.uniprot.org | 0.0008 |
| Dpy-19 like 2 GN: DPY19L2 | Proposed to serve as an anchor between the acrosome and the nucleus of the sperm. It is required for proper acrosome spreading and head formation during spermatogenesis. Pereira *et al.,* 2019 DOI: [10.1111/brv.12498](https://doi.org/10.1111/brv.12498) | 0.0021 |
| Coiled-coil domain containing 188 GN: CCDC188 | Identified as a novel candidate gene for retinitis pigmentosa. However, function remains unknown. Yi *et al.,* 2020 DOI: [10.1016/j.ebiom.2020.102792](https://doi.org/10.1016/j.ebiom.2020.102792) | 0.0033 |
| Uncharacterized protein GN: LOC110255463 | Glyco_hydro_35 domain-containing protein. Beta-galactosidase activity within the carbohydrate metabolic pathway. www.uniprot.org | 0.0034 |
| Leucine zipper transcription factor like 1 GN: LZTFL1 | Interacts with Bardet-Biedl Syndrome protein complex and regulates this complex’s ciliary traffic and signaling. Seo *et al.,* 2011 DOI: [10.1371/journal.pgen.1002358](https://doi.org/10.1371/journal.pgen.1002358) | 0.0034 |
| Peptidyl-prolyl cis-trans isomerase GN: PPIL1 | Participates in mRNA splicing, protein folding and peptidyl-prolyl isomerization. www.uniprot.org | 0.0037 |
| NmrA like redox sensor 1 GN: NMRAL1 | Transcriptional regulator which responds to cell metabolism by causing changes in gene expression. Garciandia & Suarez 2013 DOI: [10.1016/j.ydbio.2013.06.013](https://doi.org/10.1016/j.ydbio.2013.06.013) | 0.0042 |
| Glycogen synthase kinase 3 beta variant 5 GN: GSK3B | Mediates the phosphorylation of MCL1. Once phosphorylated, MCL1 undergoes ubiquitination and proteasomal degradation which releases BECLIN1 to induce axonal autophagy. Wakatsuki *et al.,* 2017 DOI: [10.1083/jcb.201606020](https://doi.org/10.1083/jcb.201606020) | 0.0048 |
| mRNA export factor GN: RAE1 | Active during interphase as a mitotic checkpoint and spindle assembly regulator. When inhibited, defects in spindle organization, chromosome alignment and segregation, and delayed cell cycle progression were observed. Lee *et al.,* 2009 DOI: [10.1111/j.1365-313X.2009.03869.x](https://doi.org/10.1111/j.1365-313x.2009.03869.x) | 0.0048 |
| Cullin 4B GN: CUL4B | E3 ubiquitin ligase believed to initiate polyubiquitination of y-tubulin and thereby control centrosomal stability through ubiquitination of y-tubulin. Thirunavukarasou *et al,* 2015 DOI: [10.1007/s11010-014-2309-7](https://doi.org/10.1007/s11010-014-2309-7) | 0.0049 |
| ACB domain-containing protein | No described function in www.uniprot.org | 0.0053 |
| Dihydroxyacetone phosphate acyltransferase GN: GNPAT | Part of the plasmalogen biosynthetic pathway. Plasmalogens make up the spermatozoa membrane and play a role in their maturation. Reisse *et al.,* 2001 DOI: [10.1095/biolreprod64.6.1689](https://doi.org/10.1095/biolreprod64.6.1689) | 0.0058 |
| 26S proteasome non-ATPase regulatory subunit 2 GN: PSMD2 | Necessary for cell proliferation and cell cycle progression. Interacts with p21 and p27 and mediates their ubiquitin-proteasomal degradation. Li *et al.,* 2018 DOI: [10.1016/j.canlet.2018.05.018](https://doi.org/10.1016/j.canlet.2018.05.018) | 0.0083 |
| Glutamine rich 2 GN: QRICH2 | Necessary for proper sperm flagellum formation. Shen *et al.,* 2019 DOI: [10.1038/s41467-018-08182-x](https://doi.org/10.1038/s41467-018-08182-x) | 0.0084 |
| Sperm acrosome membrane-associated protein 1 GN: SPACA1 | Involved in acrosome assembly, localized to the equatorial segment. Functions in sperm-egg fusion. Fujihara *et al.,* 2012 DOI: [10.1242/dev.081778](https://doi.org/10.1242/dev.081778) | 0.0087 |
| cAMP-dependent protein kinase type I-alpha regulatory subunit GN: PRKAR1A | Interacts with A-kinase anchoring proteins and responds to intracellular cAMP concentrations. Barradeau *et al.,* 2002 DOI: 10.1016/s1050-1738(02)00167-6 | 0.0106 |
| NADH dehydrogenase (ubiquinone) flavoprotein 1 mitochondrial GN: NDUFV1 | Mitochondrial protein that is part of the electron transport chain. Part of complex I. Varghese *et al.,* 2015 DOI: [10.1093/hmg/ddv344](https://doi.org/10.1093/hmg/ddv344) | 0.0107 |
| Spermatogenesis associated 3 GN: SPATA3 | Testis specific protein. Current function is unknown but believed important for spermatogenesis. Zhou *et al.,* 2020 DOI: [10.1007/s11033-019-04825-4](https://doi.org/10.1007/s11033-019-04825-4) | 0.012 |
| Actin like 7B GN: ACTL7B | A spermatogenesis-specific actin-like protein. Tanaka *et al.,* 2019 DOI: [10.22074/ijfs.2019.5702](https://doi.org/10.22074/ijfs.2019.5702) | 0.0121 |
| Cytochrome c oxidase subunit 7A2 like GN: COX7A2L | Mitochondrial respiratory chain protein. Acts as a regulatory checkpoint for Complex III, however, is nonessential for mitochondrial respiration. Lobo-Jarne *et al.,* 2018 DOI: [10.1016/j.celrep.2018.10.058](https://doi.org/10.1016/j.celrep.2018.10.058) | 0.0123 |
| Triosephosphate isomerase GN: TPI1 | Enzyme utilized during glycolysis. Catalyzes the conversion of DHAP to G3P. However, there is evidence suggesting that this is the same protein as P36, a sperm protein present along the acrosomal membrane and believed essential for normal fertilization. Auer *et al.,* 2004 DOI: [10.1002/mrd.20107](https://doi.org/10.1002/mrd.20107) | 0.0126 |
| Cilia and flagella associated protein 52 GN: CFAP52 | Involved in the motility of flagella. www.uniprot.org | 0.014 |
| SUMO peptidase family member NEDD8 specific GN: SENP8 | Contributes to proper cell cycle progression, and maintenance of neddylation levels. Coleman *et al.,* 2017 DOI: [10.7554/eLife.24325](https://doi.org/10.7554/elife.24325) | 0.0141 |
| Fibrous sheath interacting protein 2 GN: FSIP2 | Tail protein responsible for proper flagellum morphology. Martinez *et al.,* 2018 DOI: [10.1093/humrep/dey264](https://doi.org/10.1093/humrep/dey264) | 0.0144 |
| Testis specific serine kinase 4 GN: TSSK4 | Induces apoptosis in spermatogonia and spermatocytes. Seemingly functions as a quality assurance regulator of spermatogenesis. Wang *et al.,* 2015 DOI: [10.1007/s11596-015-1417-2](https://doi.org/10.1007/s11596-015-1417-2) | 0.0146 |
| Uncharacterized protein GN: LOC100626097 | No described functions www.uniprot.org | 0.0163 |
| Serpin family B member 6 GN: SERPINB6 | Serine protease inhibitor which is expressed in male germ cells and female somatic cells. Believed to play some role in gonad development, gametogenesis, and/or fertilization. Charron *et al,* 2006 DOI: [10.1002/mrd.20385](https://doi.org/10.1002/mrd.20385) | 0.0164 |
| Lon protease homolog mitochondrial GN: LONP1 | A mitochondrial protease which participates in maturation of a subset of proteins which is necessary for mitochondrial proteostasis and normal gene expression. Rendon & Shoubridge 2018 DOI: [10.1128/MCB.00412-17](https://doi.org/10.1128/mcb.00412-17) | 0.017 |
| Vacuolar protein sorting 13 homolog A GN: VPS13A | Required for lipid transfer to various organelles, including the mitochondria, where it influences morphology. Yeshaw *et al.,* 2019 DOI: [10.7554/eLife.43561](https://doi.org/10.7554/elife.43561) | 0.018 |
| Protein kinase cAMP-dependent type I regulatory subunit beta GN: PRKAR1B | Inhibits cAMP-dependent protein kinase pathways and binds the catalytic subunit of PKA www.uniprot.org | 0.0189 |
| Nardilysin convertase GN: NRD1_tv2 | Metalloendopeptidase activity. Involved in protein catabolism and regulation of membrane ectodomain proteolysis. www.uniprot.org | 0.0195 |
| Creatine kinase B-type GN: CKB | Part of the phosphocreatine biosynthetic pathway. Binds ATP and produces N-phosphocreatine. www.uniprot.org | 0.0195 |
| Uncharacterized protein GN: LOC102158372 | Integral membrane component. www.uniprot.org | 0.0203 |
| Protein phosphatase 1 regulatory subunit 9B GN: PPP1R9B | Involved in cell migration, calcium-mediated signaling and actin filament organization. www.uniprot.org | 0.0205 |
| Glutamine synthetase GN: GLUL | Enzyme which catalyzes the condensation of glutamate and ammonia to form glutamine. Cellular localization is regulated by GABA type B receptors. Huyghe *et al.,* 2014 DOI: [10.1074/jbc.M114.583534](https://dx.doi.org/10.1074%2Fjbc.M114.583534) | 0.0208 |
| BAG family molecular chaperone regulator 5 isoform b GN: BAG5_tv3 | Negatively regulates mitophagy through the suppression of Parkin recruitment. Snoo *et al.,* 2019 DOI: [10.1038/s41419-019-2132-x](https://doi.org/10.1038/s41419-019-2132-x) | 0.0212 |
| Lysozyme like 4 GN: LYZL4 | Found in high levels in the acrosome and principal piece on spermatozoa. Shown to be important for fertilization. Sun *et al.,* 2011 DOI: [10.1093/abbs/gmr017](https://doi.org/10.1093/abbs/gmr017) | 0.0226 |
| Actin related protein T3 GN: ACTRT3 | Found within male germ cell nuclei, part of the actin cytoskeleton. www.uniprot.org | 0.0228 |
| Phosphoglycerate kinase GN: PGK2 | This enzyme is specific to sperm glycolysis and replaces the PGK1 enzyme found in all other cell types. Essential for sperm motility and ATP generation. Danshina et al., 2010 DOI: [10.1095/biolreprod.109.079699](https://doi.org/10.1095/biolreprod.109.079699) | 0.0233 |
| Protein interacting with cyclin A1 GN: PROCA1 | Part of the phospholipid metabolic pathway. www.uniprot.org | 0.0263 |
| Dynein axonemal heavy chain 3 GN: DNAH3 | Known to affect sperm motility & play a role in male fertility. Rezende *et al,* 2018 DOI: [10.1111/age.12710](https://doi.org/10.1111/age.12710) | 0.0265 |
| Epididymis-specific alpha-mannosidase GN: MAN2B2 | Lysosomal type D-mannosidase found exclusively in epididymal fluid and sperm cells. Zhao *et al.,* 2019 DOI: [10.1016/j.theriogenology.2019.08.006](https://doi.org/10.1016/j.theriogenology.2019.08.006) | 0.0284 |
| Aldehyde dehydrogenase 1 family member A1 GN: ALDH1A1 | Part of the retinoic acid signaling pathway in the testes. Plays a role in spermatogenesis and responds to gonadotropin stimulation. Nourashrafeddin & Rashidi 2018 PMID: **29436793** | 0.029 |
| Translocase of outer mitochondrial membrane 34 GN: TOMM34 | A chaperone-like protein which imports preproteins into the mitochondria. Blesa *et al,* 2008 DOI: [10.1139/o07-151](https://doi.org/10.1139/o07-151) | 0.0306 |
| Kinetochore localized astrin (SPAG5) binding protein GN: KNSTRN | Involved in chromosome segregation, regulation of attachment of spindle microtubules to kinetochore, & spindle organization. www.uniprot.org | 0.033 |
| Testin GN: TES | A protein which is secreted by Sertoli cells and regulates the blood-testis barrier. Also found in ovaries. Su *et al.,* 2020 DOI: [10.1002/jcp.29541](https://doi.org/10.1002/jcp.29541) | 0.0332 |
| Family with sequence similarity 170 member A GN: FAM170A | No described function. www.uniprot.org | 0.0804 |
| SAMM50 sorting and assembly machinery component GN: SAMM50 | Regulator of PINK1-Parkin mediated mitophagy. A Samm50 depletion results in PINK1 build up, Parkin recruitment and subsequent mitophagy. Jian *et al.,* 2018 DOI: [10.1016/j.celrep.2018.05.015](https://doi.org/10.1016/j.celrep.2018.05.015) | 0.0345 |
| Fascin GN: FSCN3 | Essential for spermatid development, establishment and maintenance of cell polarity, actin filament binding. Expressed in the spermatid head. Tubb *et al.,* 2002 DOI: [10.1006/excr.2002.5486](https://doi.org/10.1006/excr.2002.5486) | 0.0347 |
| Tyrosine 3-monooxygenase/tryptophan 5-monooxygenase activation protein zeta | Newly described regulator of capacitation-associated tyrosine phosphorylation. Saez *et al.,* 2019 DOI: [10.1111/andr.12634](https://doi.org/10.1111/andr.12634) | 0.0379 |
| NSF attachment protein alpha GN: NAPA | Soluble N-ethylmaleimide-sensitive factor attaching activities. www.uniprot.org | 0.0384 |
| WD repeat domain 63 GN: WDR63 | Upregulated by p53, negatively regulates cell migration, invasion, and metastasis. Zhao *et al,* 2020 DOI: [10.15252/embr.201949269](https://doi.org/10.15252/embr.201949269) | 0.0422 |
| Uncharacterized protein GN: LOC100154312 | Involved with the centrosomes, but no described function. www.uniprot.org | 0.0429 |
| Zona pellucida-binding protein 1 GN: ZPBP | Important for acrosome assembly, as well as binding of sperm to the zona pellucida. Yatsenko *et al.,* 2012 DOI: [10.1093/molehr/gar057](https://doi.org/10.1093/molehr/gar057) | 0.043 |
| Mitochondrial associated cysteine-rich protein GN: SMCP | Localized to the mitochondrial capsule and contributes to flagellum movement. Hawthorne *et al.,* 2006 DOI: [10.1016/j.ygeno.2005.09.010](https://doi.org/10.1016/j.ygeno.2005.09.010) | 0.0436 |
| Uncharacterized protein GN: SCO2 | Induces p53-mediated apoptosis. It is a cytochrome c oxidase assembly factor and increases reactive oxygen species production. Madan *et al.,* 2013 DOI: [10.1128/MCB.06798-11](https://doi.org/10.1128/mcb.06798-11) | 0.0438 |
| Epididymal sperm-binding protein 1 GN: ELSPBP1 | In bovine, this protein binds dead sperm within the epididymis. D’Amours *et al.* 2012 DOI: [10.1095/biolreprod.112.100990](https://doi.org/10.1095/biolreprod.112.100990); | 0.0445 |
| Armadillo repeat containing 4 GN: ARMC4 | Participates in cilium movement and outer dynein arm assembly. www.uniprot.org | 0.0448 |
| Chromosome 1 open reading frame 56 GN: C1orf56 | Regulates cell population proliferation. www.uniprot.org | 0.0457 |
| Regulator of G protein signaling 22 GN: RGS22 | Regulates G-protein signaling during spermiogenesis. Hu *et al.,* 2008 DOI: [10.1095/biolreprod.107.067504](https://doi.org/10.1095/biolreprod.107.067504) | 0.0457 |
| Hypoxanthine-guanine phosphoribosyltransferase GN: HPRT1 | Plays a role in purine regulation and generation through the salvage of IMP and GMP. Appears to be found in high amounts within many types of cancer cells Townsend *et al.,* 2018. DOI: [10.1007/s12032-018-1144-1](https://doi.org/10.1007/s12032-018-1144-1) | 0.0468 |
| Kinesin-like protein GN: KIF9 | Testis specific kinesin. Regulates flagellar motility and acts as a molecular motor in spermatozoa tails. Miyata *et al.,* 2020 DOI: [10.1096/fj.201902755R](https://doi.org/10.1096/fj.201902755r) | 0.0468 |
| Immunity-related GTPase family cinema protein GN: IRGC | GTPase activity, located in the cell membrane. [www.uniprot.org](http://www.uniprot.org) | 0.0476 |
| Dynein axonemal heavy chain 17 GN: DNAH17 | Sperm-specific axonemal outer dynein arm protein. Necessary for male fertility and normal flagellum movement and morphology. Whitfield *et al.,* 2019 DOI: [10.1016/j.ajhg.2019.04.015](https://doi.org/10.1016/j.ajhg.2019.04.015) | 0.0478 |
| Vaccinia related kinase 3 GN: VRK3 | Part of the Wnt signaling pathway, negatively regulates ERK1 & 2, and positively regulates phosphoprotein phosphatase activity. www.uniprot.org | 0.0488 |
| Dynein axonemal heavy chain 8 GN: DNAH8 | Sperm-specific axonemal outer dynein arm protein. Necessary for male fertility and normal flagellum movement and morphology. Whitfield *et al.,* 2019 DOI: [10.1016/j.ajhg.2019.04.015](https://doi.org/10.1016/j.ajhg.2019.04.015) | 0.0498 |
| Retinoic acid receptor responder 1 GN: RARRES1 | Metalloendopeptidase inhibitor activity, found in the extracellular matrix. www.uniprot.org | 0.0505 |
| NADH:ubiquinone oxidoreductase subunit A9 GN: NDUFA9 | Subunit required for complex I assembly in the electron transport chain. Mitochondrial protein. Baertling *et al.,* 2018 DOI: [10.1111/cge.13089](https://doi.org/10.1111/cge.13089) | 0.051 |
| SEC13 homolog nuclear pore and COPII coat complex component GN: SEC13 | Involved in COPII-coated vesicle budding and protein transport from the ER. www.uniprot.org | 0.0512 |
| Uncharacterized protein GN: LOC100514982 | No described function www.uniprot.org | 0.0514 |
| L-lactate dehydrogenase C GN: LDHC | Testis-specific isozyme found in male germ cells. Localizes to the principal piece in mature sperm but found in all spermatogenic cells. Appears to be required for fertilization. Goldberg *et al.,* 2010 DOI: [10.2164/jandrol.109.008367](https://doi.org/10.2164/jandrol.109.008367) | 0.0523 |
| TBC1 domain family member 21 GN: TBC1D21 | Localized to elongating spermatids during spermatogenesis, this protein appears to bind Rap1 (a protein crucial for proper spermatogenesis). Ke *et al.,* 2018 DOI: [10.3390/ijms19113292](https://doi.org/10.3390/ijms19113292) | 0.0567 |
| Uncharacterized protein GN: LOC100515166 | IF rod domain-containing protein. Structural protein, part of the intermediate filaments. www.uniprot.org | 0.0594 |
| Uncharacterized protein GN: LOC100522130 | Hydrolase activity utilizes magnesium as a cofactor. www.uniprot.org | 0.0636 |
| Actin like 9 GN: ACTL9 | Part of the actin-based cytoskeleton. www.uniprot.org | 0.065 |
| NADH dehydrogenase (ubiquinone) iron-sulfur protein 6 mitochondrial GN: NDUFS6 | Subunit of Complex I in the electron transport chain. Mitochondrial protein necessary for normal Complex I assembly. Kirby *et al,* 2004 DOI: [10.1172/JCI20683](https://doi.org/10.1172/jci20683) | 0.0655 |
| Uncharacterized protein GN: LOC102164346 | FAM75 domain-containing protein. No characterized functions. [www.uniprot.org](http://www.uniprot.org) | 0.0665 |
| Schlafen like 1 GN: SLFNL1 | Testis-abundant protein. Shown to not be essential for male fertility. Park *et al,* 2020 DOI: [10.1093/biolre/ioaa084](https://doi.org/10.1093/biolre/ioaa084) | 0.0679 |
| Dynein regulatory complex subunit 7 GN: DRC7 | Required for flagellum formation and male fertility. Morohoshi *et al.,* 2020 DOI: [10.1371/journal.pgen.1008585](https://doi.org/10.1371/journal.pgen.1008585) | 0.0693 |
| Uncharacterized protein GN: PSMC2 | Proteasome 26S subunit ATPase 2. Positively regulates RNA polymerase II complex assembly and participates in ubiquitin-dependent protein catabolism. www.uniprot.org | 0.0694 |
| Uncharacterized protein GN: PDHB | Pyruvate dehydrogenase B. Part of the pyruvate to acetyl-CoA pathway. www.uniprot.org | 0.0701 |
| Coiled-coil-helix-coiled-coil-helix domain containing 5 GN: CHCHD5 | No characterized function. www.uniprot.org | 0.0707 |
| Actin related protein T2 GN: ACTRT2 | Located in the post-acrosomal region and mid-piece of the sperm. Declines in this protein reduce sperm motility. Liu *et al,* 2015 DOI: [10.1111/andr.289](https://doi.org/10.1111/andr.289) | 0.0714 |
| Keratin 4 GN: KRT4 | Part of the keratin filament-based components of the cytoskeleton. www.uniprot.org | 0.0714 |
| Ubiquitin thioesterase a.k.a. OTU deubiquitinase GN: OTUB1 | Known deubiquitinating enzyme. Shown to regulate p53 and promote mitochondria-mediated apoptosis. Chen *et al.,* 2017 DOI: [10.18632/oncotarget.14278](https://doi.org/10.18632/oncotarget.14278) | 0.0716 |
| Rhophilin associated tail protein 1 like GN: ROPN1L | Sperm tail protein necessary for normal tail morphology and motility in sperm. Rattanachan *et al.,* 2014 DOI: [10.1016/j.actatropica.2014.08.002](https://doi.org/10.1016/j.actatropica.2014.08.002) | 0.0716 |
| HYDIN axonemal central pair apparatus protein GN: HYDIN | Ciliary protein associated with heart tissue. Involved in GATA4 expression within heart tissues. Cao *et al.,* 2020 DOI: [10.1016/j.mod.2020.103611](https://doi.org/10.1016/j.mod.2020.103611) | 0.0725 |
| Heat shock 70 kDa protein 1-like GN: HSPA1L | Chaperone to HSP70 and found on the sperm plasma membrane. Naaby-Hansen & Herr 2010 DOI: [10.1016/j.jri.2009.09.006](https://doi.org/10.1016/j.jri.2009.09.006) | 0.0727 |
| Actin like 7A GN: ACTL7A | A spermatogenesis-specific actin like protein. Tanaka *et al.,* 2019 DOI: [10.22074/ijfs.2019.5702](https://doi.org/10.22074/ijfs.2019.5702) | 0.0729 |
| T-complex protein 1 subunit gamma GN: CCT3 | The t-complex protein 1 is a complex within cells which is responsible for protein folding. It is made up of 8 distinct subunits. Believed to interact with Zona-binding protein 2 and participate in sperm-zona binding. Dun *et al.,* 2011 DOI: [10.1074/jbc.M110.188888](https://doi.org/10.1074/jbc.m110.188888) | 0.0302 |
| Uncharacterized protein Accession: A0A287AEH4 | DUF4599 domain-containing protein. Membrane component protein. www.uniprot.org | 0.0759 |
| Phosphatidylethanolamine-binding protein 4 GN: pebp4 | A secreted protein, unlike pebp1, 2, & 3. Inhibits ERK from activating EGF. Believed to participate in other pathways as well. He *et al,* 2016 DOI: [10.1016/j.bbamcr.2016.03.022](https://doi.org/10.1016/j.bbamcr.2016.03.022) | 0.0764 |
| Acyl-CoA synthetase long chain family member 6 GN: ACSL6 | Required form normal spermatogenesis in mice. Highly expressed in spermatids. Shishikura *et al.,*2019 DOI: [10.1096/fj.201901074R](https://doi.org/10.1096/fj.201901074r) | 0.0774 |
| NADH-cytochrome b5 reductase GN: CYB5R1 | Flavoprotein involved in transferring reducing equivalents from NADH using FAD. Elahian *et al,* 2012 DOI: [10.3109/07388551.2012.732031](https://doi.org/10.3109/07388551.2012.732031) | 0.0802 |
| Calmodulin 2 | Calcium-mediated signaling & calcium ion binder. In stallion sperm, calmodulin plays a role in regulating sperm motility. Lasko *et al.,* 2012 DOI: [10.1016/j.anireprosci.2012.05.007](https://doi.org/10.1016/j.anireprosci.2012.05.007) | 0.0804 |
| Dynein axonemal heavy chain 7 GN: DNAH7 | Involved in inner dynein arm assembly and cilium movement. www.uniprot.org | 0.084 |
| NADH dehydrogenase (ubiquinone) 1 subunit C2 GN: NDUFC2 | Needed for proper mitochondrial function. Part of complex I in the electron transport chain of the mitochondria. Raffa *et al.,* 2019 DOI: [10.1016/j.ijcard.2019.02.027](https://doi.org/10.1016/j.ijcard.2019.02.027) | 0.0842 |
| FUN14 domain-containing protein 2 GN: FUNDC2 | FUNDC2 binds PIP3 and works through the AKT1/BAD/ BCL2L1 axis on the mitochondrial outer membrane. BCL2L1 protects cells against apoptotic hypoxia stress. A removal of FUNDC2 would coincide with a loss of stress protection and an increase in apoptosis and possibly mitophagy. Ma *et al.,* 2018 DOI: [10.1038/s41418-018-0121-8](https://doi.org/10.1038/s41418-018-0121-8) | 0.0845 |
| Dynein axonemal intermediate chain 2 GN: DNAI2 | Plays a role in cilium movement, & outer dynein arm assembly. www.uniprot.org | 0.0884 |
| A-kinase anchoring protein 3 GN: AKAP3 | Essential for sperm motility, capacitation, and the acrosome reaction. Degraded by tyrosine phosphorylation during capacitation. Vizel *et al.,* 2015 DOI: [10.1016/j.bbagen.2015.06.005](https://doi.org/10.1016/j.bbagen.2015.06.005) | 0.0886 |
| Uncharacterized protein Accession: A0A287BR23 | DUF4685 domain-containing protein. No characterized function. www.uniprot.org | 0.0914 |
| Angiotensin-converting enzyme GN: ACE | Zinc metallopeptidase, membrane protein. Important for sperm transport through the oviduct and sperm-zona pellucida binding. Likely involved in the distribution of ADAM3 for zona binding. Yamaguchi *et al.,* 2006 DOI: [10.1095/biolreprod.106.052977](https://doi.org/10.1095/biolreprod.106.052977) | 0.094 |
| Uncharacterized protein Accession: K7GM09 | Integral component of membrane. Protein appears like: Calponin-homology domain-containing protein. www.uniprot.org | 0.0956 |
| Outer dense fiber protein 1 GN: ODF1 | Sperm structural protein, crucial for proper head attachment and normal connecting piece morphology. Schneider *et al.,* 2016 DOI: [10.1074/mcp.M116.060343](https://doi.org/10.1074/mcp.m116.060343) | 0.0959 |
| Family with sequence similarity 71 member D GN: FAM71D | Associated with sperm motility and is found in sperm flagella. Ma *et al.,* 2017 DOI: [10.1093/humrep/dex290](https://doi.org/10.1093/humrep/dex290) | 0.0962 |
| Chromosome 3 open reading frame 84 GN: C3orf84 | HDNR domain-containing protein. No described function. www.uniprot.org | 0.0977 |
| NADH:ubiquinone oxidoreductase subunit A7 GN: NDUFA7 | Mitochondrial protein, part of Complex I in the electron transport chain. Alston *et al.,* 2018 DOI: [10.1016/j.ajhg.2018.08.013](https://doi.org/10.1016/j.ajhg.2018.08.013) | 0.0983 |
| Glycerol kinase GN: GK | Converts glycerol to glycerol-3-phosphate. www.uniprot.org | 0.0985 |
