# Supplementary material for: Identification of candidate mitochondrial inheritance determinants using the mammalian cell-free system
Source: eLife. 2023 Jul 20;12:RP85596. doi: 10.7554/eLife.85596 (PMC10393022; doi:10.7554/eLife.85596)
Supplement: Supplementary file 3. [file elife-85596-supp3.docx]

**Supplementary file 3:** Proteomic identification of mitophagy and sperm remodeling cofactors in porcine cell-free system after 24 hours of co-incubation.

| Protein of Interest  Legend:  GN = Gene name  PE = protein existence level  SV = Sequence version  (PE and SV only included for proteins which had multiple versions in the table) | Proposed role in autophagy/mitophagy/post-fertilization sperm structure remodeling by zygote, and original references describing identified proteins’ role in those events. | P value based off 3 rep T-test analysis |
| --- | --- | --- |
| **CLASS 1 – Proteins detectable on spermatozoa only after extract exposure.** | | |
| Zona pellucida glycoprotein 3 GN: ZP3 | Involved in sperm-egg recognition, acrosomal reaction, and polyspermy defense. Zimmerman *et al.,* 2011 DOI: [10.1371/journal.pone.0017256](https://dx.doi.org/10.1371%2Fjournal.pone.0017256) | 0.0001 |
| Metalloendopeptidase a.k.a. Ovastacin GN: ASTL | Member of peptidase family involved in reproductive protein processing in *Drosophila;* (ov)astacin is secreted by mouse oocyte to cleave zona, prevent polyspermy. Ram *et al.,* 2006 DOI: [10.1073/pnas.0606228103](https://doi.org/10.1073/pnas.0606228103) ; Burkart *et al.,* 2012; DOI: [10.1083/jcb.201112094](https://doi.org/10.1083/jcb.201112094) | 0.0002 |
| Peroxiredoxin 3 GN: PRDX3 | Necessary for normal mitochondrial function. Localizes to the mitochondria after peroxide exposure and acts as a peroxide reducing agent. Wonsey *et al.,* 2002 DOI: [10.1073/pnas.102523299](https://doi.org/10.1073/pnas.102523299) | 0.001 |
| Zona pellucida glycoprotein 4 GN: ZP4 | Involved in sperm-egg recognition, acrosomal reaction, and polyspermy defense. Zimmerman *et al.,* 2011 DOI: [10.1371/journal.pone.0017256](https://dx.doi.org/10.1371%2Fjournal.pone.0017256) | 0.1837 |
| Small Calcium-binding mitochondrial carrier 1 GN: SCAMC | Protein involved in ATP-Mg/Pi transport across the inner mitochondrial membrane, in a calcium regulated manner. Arco & Satrustegui 2004 DOI: [10.1074/jbc.M401417200](https://doi.org/10.1074/jbc.m401417200) | 0.1839 |
| Lactamase beta GN: LACTB | Localized to the intermembrane space of the mitochondria. Forms long, stable filaments to create intramitochondrial membrane organization and micro-compartments within the mitochondria. Polianskyte *et al.,* 2009 DOI: [10.1073/pnas.0906734106](https://doi.org/10.1073/pnas.0906734106) | 0.1844 |
| Nucleophosmin/nucleoplasmin 2 GN: NPM2 | Sperm nucleus remodeling after fertilization. Required for histone import into paternal pronucleus. McLay & Clark., 2003 DOI: [10.1530/rep.0.1250625](https://doi.org/10.1530/rep.0.1250625) | 0.1861 |
| Zona Pellucida glycoprotein 2 GN: ZP2 | Involved in sperm-egg recognition, acrosomal reaction, and polyspermy defense. Zimmerman *et al.,* 2011 DOI: [10.1371/journal.pone.0017256](https://dx.doi.org/10.1371%2Fjournal.pone.0017256) | 0.1875 |
| Peptidyl arginine deiminase 6 GN: PADI6 | Essential for early zygotal development, specifically cytoskeletal sheet formation through citrullination. Essential for female fertility. Esposito *et al.,* 2007 DOI: [10.1016/j.mce.2007.05.005](http://doi.org/10.1016/j.mce.2007.05.005) | 0.1875 |
| Major Vault Protein GN: MVP | Regulates cell growth and survival through the STAT3 and Akt signaling pathways. Das *et al.,* 2016 DOI: [10.1016/j.cellsig.2015.10.007](https://doi.org/10.1016/j.cellsig.2015.10.007) | 0.1889 |
| **CLASS 2 - Detected in the vehicle and primed control spermatozoa, increased in extract-exposed spermatozoa.** | | |
| High mobility group box 3 GN: HMGB3 | Positively regulates transcription through RNA polymerase II. Located in the nucleus. www.uniprot.org | 0.0039 |
| Myeloid leukemia factor 1 GN: MLF1 | Negatively regulates the HOP mitochondrial protein complex to regulate cell survival. Serves as a proapoptotic antagonist. Sun *et al.,* 2017 DOI: [10.1016/j.bbamcr.2017.01.016](https://doi.org/10.1016/j.bbamcr.2017.01.016) | 0.005 |
| PHD finger protein 7 GN: PHF7 | Plays a role in histone-to-protamine exchange during spermiogenesis. Appears to begin the ubiquitylation of H2A in round spermatids. Wang *et al.,* 2019 DOI: [10.1242/dev.175547](https://doi.org/10.1242/dev.175547) | 0.0063 |
| Spermatogenesis associated 24 GN: SPATA24 | DNA binding protein. Located in cytosol and nucleus. www.uniprot.org | 0.0122 |
| Chromosome 12 open reading frame 100 GN: C12H17orf100 | No characterized function www.uniprot.org | 0.0315 |
| Ubiquitin specific peptidase 50 GN: USP50 | A deubiquitinating enzyme which regulates the cell cycle through prevention of Wee1 degradation. USP50 interacts with HSP90 and accumulates after DNA damage. It’s believed to prevent entry into mitosis following DNA damage. Aressy *et al.,* 2010 DOI: [10.4161/cc.9.18.13133](https://doi.org/10.4161/cc.9.18.13133) | 0.0333 |
| Proteasome subunit alpha type GN: PSMA8 | A testis specific proteasomal subunit found during spermatogenesis. Appears crucial for proper meiotic exit by the forming spermatids. Gomez-H *et al.,* 2019 DOI: [10.1371/journal.pgen.1008316](https://doi.org/10.1371/journal.pgen.1008316) | 0.046 |
| Chromosome 2 open reading frame 70 GN: C2orf70 | No characterized function www.uniprot.org | 0.0487 |
| Theg spermatid protein GN: THEG | Testicular haploid expressed gene. Thought to only be expressed in spermatid cells. Though not essential for spermatogenesis it is thought to participate in Sertoli cell and spermatid interactions. Mannan *et al.,* 2003 DOI: [10.1095/biolreprod.103.017400](https://doi.org/10.1095/biolreprod.103.017400) | 0.0872 |
| Proteasome assembly chaperone 2 GN: PSMG2 | Interacts closely with PAC1 and they help ensure proper proteasome core formation, acting as a quality control mechanism. Wu *et al.,* 2018 DOI: [10.1111/gtc.12631](https://doi.org/10.1111/gtc.12631) | 0.0878 |
| **CLASS 3 – Proteins detected in spermatozoa before extract, reduced after extract exposure.** | | |
| Creatine Kinase B-type GN: CKB | Part of the phosphocreatine biosynthetic pathway. Binds ATP and produces N-phosphocreatine. www.uniprot.org | 0.000043 |
| Platelet-activating factor acetylhydrolase IB subunit alpha GN: PAFAH1B1 | Plays a role in spermatogenesis and is found in the acrosome and midpiece of spermatozoa. However, it is also found in the membrane of oocytes and zygotes. Yao *et al,* 2015 DOI: [10.1016/j.rbmo.2015.07.010](https://doi.org/10.1016/j.rbmo.2015.07.010) | 0.0039 |
| Electron transfer flavoprotein subunit beta GN: ETFB | Mitochondrial protein that is part of the electron transport chain. Serves as an electron acceptor molecule. Schmiesing *et al,* 2014 DOI: [10.1371/journal.pone.0087715](https://doi.org/10.1371/journal.pone.0087715) | 0.0121 |
| Chromosome 17 open reading frame 98 GN: C17orf98 | No characterized function. www.uniprot.org | 0.0121 |
| Glutamate-cysteine ligase modifier subunit GN: GCLM | Regulates mitochondrial depolarization and participates in the glutathione biosynthetic pathway. www.uniprot.org | 0.0125 |
| Serotransferrin GN: TF | Modulates interactions between endosomes and mitochondria. Das *et al.,* 2016 DOI: [10.1083/jcb.201602069](https://doi.org/10.1083/jcb.201602069) | 0.0177 |
| L-lactate dehydrogenase B chain GN: LDHB | When deacetylated by SIRT5, autophagy is promoted. Shi *et al.,* 2019 DOI: [10.1002/1878-0261.12408](https://doi.org/10.1002/1878-0261.12408) | 0.0184 |
| Sperm-associated acrosin inhibitor isoform X1 GN: LOC396905 | [Kazal](http://Kazal) type serine proteinase inhibitor. | 0.0199 |
| Receptor of activated protein C kinase 1 GN: RACK1 | Ribosomal protein located on the small ribosomal subunit and appears to regulate translation. Gallo *et al.,* 2018 DOI: [10.1128/MCB.00230-18](https://doi.org/10.1128/mcb.00230-18) | 0.0203 |
| Radial spoke head 14 homolog GN: RSPH14 | No characterized function. [www.uniprot.org](http://www.uniprot.org) | 0.0218 |
| Eukaryotic translation initiation factor 4E GN: EIF4E | Reduces an apoptotic susceptibility via inhibition of mitochondrial cytochrome c release. Li *et al.,* 2003 DOI: [10.1074/jbc.M208821200](https://doi.org/10.1074/jbc.m208821200) | 0.024 |
| Lactotransferrin GN: LTF | Iron-binding glycoprotein. Regulates iron absorption, & immune responses. Hao *et al.,* 2019 DOI: [10.2174/1389203719666180514150921](https://doi.org/10.2174/1389203719666180514150921) | 0.0279 |
| Transducin beta like 2 GN: TBL2 | Localizes to the endoplasmic reticulum and interacts with the 60S ribosomal subunit. Also works with PERK during ER stress. Tsukumo *et al,* 2015 DOI: [10.1016/j.bbrc.2015.04.144](https://doi.org/10.1016/j.bbrc.2015.04.144) | 0.0312 |
| Histone H2A GN: HIST2H2AB | A histone 2A variant which confers specific properties to the nucleosome. Plays a dominant role in chromatin dynamics and function. Osakabe *et al.,* 2018 DOI: [10.1093/nar/gky540](https://doi.org/10.1093/nar/gky540) | 0.0342 |
| Adenylate cyclase 3 GN: ADCY3 | Required for normal spermatozoa function. Plays a role in male fertility and appears to localize to the acrosomal membrane, may play a role in the acrosomal reaction. Livera *et al.,* 2005 DOI: [10.1210/me.2004-0318](https://doi.org/10.1210/me.2004-0318) | 0.0358 |
| Retinoic acid receptor responder 1 GN: RARRES1 | Metalloendopeptidase inhibitor activity, found in the extracellular matrix. www.uniprot.org | 0.0401 |
| Spermatogenesis-associated protein 20 GN: SPATA20 | Participates in DNA damage response in male germ cells. Facilitates DNA repair. Zheng *et al.,* 2017 DOI: [10.1371/journal.pone.0178535](https://doi.org/10.1371/journal.pone.0178535) | 0.0469 |
| ADP ribosylation factor like GTPase 1 GN: ARL1 | Involved in endosomal trans-Golgi network and secretory traffic, lipid droplet formation, innate immunity, stress tolerance and unfolded protein response. Yu & Lee 2017 DOI: [10.1242/jcs.201319](https://doi.org/10.1242/jcs.201319) | 0.0536 |
| Glycerol-3-phosphate dehydrogenase (NAD[+]) GN: GPD1L | Converts glycerol 3-phosphate into dihydroxyacetone phosphate. Part of the carbohydrate metabolic process. www.uniprot.org | 0.0563 |
| G protein subunit beta 2 GN: GNB2 | Binds the G-protein gamma-subunit & GTPases. Part of the G protein-coupled receptor signaling pathway. www.uniprot.org | 0.0593 |
| IQ motif containing F2 GN: IQCF2 | Binds calmodulin. www.uniprot.org | 0.0623 |
| RAB3B member RAS oncogene family GN: RAB3B | Involved in various cellular pathways. Has GTPase activity and binds myosin. www.uniprot.org | 0.0634 |
| Membrane spanning 4-domains A14 GN: MS4A14 | Found during spermatogenesis in round spermatids to spermatozoa. Localized to the acrosome and midpiece. Involved in fertilization and zygotic division. Xu *et al.,* 2014 DOI: [10.1530/REP-14-0087](https://doi.org/10.1530/rep-14-0087) | 0.0643 |
| Keratin 14 GN: KRT14 | Part of the intermediate filament portion of the cytoskeleton. www.uniprot.org | 0.0673 |
| Endosome associated trafficking regulator 1 GN: ENTR1 | Controls Fas-mediated apoptotic signaling via binding to Dysbindin. Sharma *et al.,* 2019 DOI: [10.1038/s41467-019-11025-y](https://doi.org/10.1038/s41467-019-11025-y) | 0.0691 |
| NME/NM23 family member 5 GN: NME5 | Involved in spermatid development, flagellum assembly, and acts as a nucleoside diphosphate kinase. [www.uniprot.org](http://www.uniprot.org) | 0.07 |
| Deoxythymidylate kinase GN: DTYMK | Part of the mitochondrial nucleotide salvage pathway. Mutations in this gene cause mitochondrial DNA depletion syndrome. Lam *et al.,* 2019 DOI: [10.1016/j.cca.2019.06.028](https://doi.org/10.1016/j.cca.2019.06.028) | 0.0714 |
| Rho GDP dissociation inhibitor alpha GN: ARHGDIA | Regulates protein localization, part of the Rho and semaphoring-plexin signaling pathways. www.uniprot.org | 0.0722 |
| Thioredoxin domain containing 17 GN: TXNDC17 | Peroxidase and protein-disulfide reductase activities. www.uniprot.org | 0.0756 |
| Keratin 7 GN: KRT7 | Part of the keratin-based cytoskeleton. www.uniprot.org | 0.0777 |
| UPAR/Ly6 domain-containing protein GN: LYPD6 | No characterized function in . www.uniprot.org | 0.0796 |
| Uncharacterized protein GN: LOC100738961 | Probable GPI-anchored adhesin-like protein PGA55 | 0.0827 |
| GTP-binding protein SAR1a GN: SAR1A | Part of the COPII-coated vesicle transporting system. GTPase activity. www.uniprot.org | 0.0834 |
| ATP binding cassette subfamily E member 1 GN: ABCE1 | Undergoes ubiquitination by NOT4 in response to mitochondrial damage. Acts as translational quality control. Showing that PINK1-directed mitophagy influences translation in cells. We *et al.,* 2018 DOI: [10.1016/j.cmet.2018.05.007](https://doi.org/10.1016/j.cmet.2018.05.007) | 0.0856 |
| Sarcoplasmic/endoplasmic reticulum calcium ATPase 2 GN: ATP2A2 | Plays roles in calcium ion homeostasis, organelle organization, response to oxidative stress and ER transport. www.uniprot.org | 0.0876 |
| Keratin, type I cytoskeletal 13 GN: LOC100515166 | IF rod domain-containing protein. A structural protein, part of the intermediate filaments. www.uniprot.org | 0.0891 |
| 60S acidic ribosomal protein P0 GN: RPLP0 | When depleted, PLAAT4 induced cell cycle arrest and cell death are increased. Wang *et al.,* 2019 DOI: [10.1007/s12013-019-00876-3](https://doi.org/10.1007/s12013-019-00876-3) | 0.0891 |
| Tctex-type 1 dynein light chain GN: DYNLT1 | Necessary for male germ cell development. Known to be involved in protein trafficking, membrane vesiculation, cell cycle regulation and stem cell differentiation. Indu *et al.,* 2015 DOI: [10.1074/mcp.M115.050005](https://doi.org/10.1074/mcp.m115.050005) | 0.0959 |
| Glutathione transferase Accession: F1SL35 | Part of the glutathione metabolic process. www.uniprot.org | 0.0977 |
| Eukaryotic translation elongation factor 2 GN: EEF2 | Catalyzes reverse localization of the ribosome. Susorov *et al.,* 2018 DOI: [10.1074/jbc.RA117.000761](https://doi.org/10.1074/jbc.ra117.000761) | 0.0982 |
| Zymogen granule protein 16B GN: ZG16B | Depletion increases aneuploidy, promotes apoptosis, and activates the Wnt/Beta-catenin pathway. Escudero-Paniagua *et al.,* 2020 DOI: [10.1093/carcin/bgz093](https://doi.org/10.1093/carcin/bgz093) | 0.0991 |
| Keratin 8 GN: KRT8 | When phosphorylated, this protein induces autophagy impairment by affecting the fusion of autophagosomes and lysosomes. Miao *et al.,* 2020 DOI: [10.1111/jcmm.14998](https://doi.org/10.1111/jcmm.14998) | 0.0994 |
| NADH:ubiquinone oxidoreductase core subunit S8 GN: NDUFS8 | Part of complex I in the mitochondrial respiratory chain. www.uniprot.org | 0.0997 |
